# Diagnostic Accuracy of Endometrial Aspiration Cytology Compared With Histopathology in Abnormal Uterine Bleeding: A Cross-Sectional Study at a Tertiary Care Hospital

**DOI:** 10.7759/cureus.115891

**Published:** 2026-09-07

**Authors:** Shivam U More, Sabiha Maimoon, Trupti A Dongre, Payal B Nagargoje

**Affiliations:** 1 Pathology, N.K.P. Salve Institute of Medical Sciences and Lata Mangeshkar Hospital, Nagpur, IND

**Keywords:** abnormal uterine bleeding, diagnostic accuracy, endometrial aspiration cytology, endometrial carcinoma, endometrial hyperplasia, histopathology

## Abstract

Introduction

Abnormal uterine bleeding (AUB) is a frequent indication for endometrial evaluation. Histopathological examination remains the diagnostic reference standard, but tissue sampling by curettage or biopsy may be invasive and resource-intensive. Endometrial aspiration cytology (EAC) offers a rapid, minimally invasive alternative that can provide an early impression of endometrial pathology. This study assessed the diagnostic performance of EAC against histopathology in women presenting with AUB.

Materials and methods

This prospective cross-sectional study was conducted from January 2024 to December 2025 in the Department of Pathology, N.K.P. Salve Institute of Medical Sciences & Research Centre and Lata Mangeshkar Hospital, Nagpur, India. Data collection was carried out from May 2024 to August 2025. A total of 100 women with clinically diagnosed AUB were enrolled. Endometrial aspirates were prepared as alcohol-fixed smears and stained with Papanicolaou and hematoxylin and eosin stains. Cytomorphological findings were categorized and correlated with the corresponding histopathological diagnosis. Diagnostic performance was expressed as sensitivity, specificity, positive predictive value (PPV), negative predictive value (NPV), and overall accuracy. Associations with age, bleeding pattern, and ultrasonographic endometrial thickness were assessed statistically.

Results

The largest age group was 41-50 years, comprising 38 (38%) participants, followed by 31-40 years with 32 (32%). Menorrhagia was the most frequent presentation in 36 (36%) participants. On ultrasonography, 46 (46%) participants had an endometrial thickness of 5-10 mm and 38 (38%) had a thickness greater than 10 mm. Secretory endometrium was the most common cytological diagnosis in 38 (38%) and histopathological diagnosis in 37 (37%), followed by proliferative endometrium in 35 (35%) and 34 (34%), respectively. Histopathology identified simple glandular hyperplasia without atypia in 10 (10%), hyperplasia with atypia in nine (9%), endometrial carcinoma in nine (9%), and granulomatous pathology in one (1%). Cytological findings correlated strongly with histopathology (Pearson chi-square = 132.24, p < 0.001). Postmenopausal bleeding and increasing endometrial thickness were significantly associated with premalignant and malignant pathology. EAC showed a sensitivity of 45/50 (90.0%), specificity of 44/50 (88.0%), PPV of 45/51 (88.2%), NPV of 44/49 (89.8%), and overall diagnostic accuracy of 89/100 (89.0%).

Conclusions

EAC showed good diagnostic performance and close correlation with histopathology in women with AUB. Its speed, simplicity, and minimally invasive nature make it useful as a screening and preliminary diagnostic procedure, particularly where access to operative sampling is limited. Histopathology remains essential for definitive classification, especially when atypia or malignancy is suspected.

## Introduction

Abnormal uterine bleeding (AUB) is one of the most common reasons for gynecological consultation and may occur across the reproductive, perimenopausal, and postmenopausal years. The term covers disturbances in the frequency, regularity, duration, or volume of uterine bleeding and is best regarded as a clinical presentation rather than a diagnosis in itself. The International Federation of Gynecology and Obstetrics (FIGO) PALM-COEIN system provides a practical framework for separating structural causes from non-structural causes of AUB [1].

Although many women with AUB have benign cyclical or hormonal changes, the diagnostic spectrum also includes endometrial hyperplasia, atypical hyperplasia/endometrioid intraepithelial neoplasia, and endometrial carcinoma. The possibility of premalignant or malignant disease becomes particularly important with advancing age and in women with postmenopausal bleeding. For that reason, a reliable assessment of the endometrium is central to the work-up of persistent or unexplained AUB [2,3].

Histopathological examination (HPE) of endometrial tissue obtained by biopsy, curettage, or hysteroscopy-guided sampling remains the reference standard because it permits evaluation of both cytological detail and tissue architecture. However, conventional tissue sampling may require cervical dilatation, anesthesia, an operating-room or procedure-room setting, and additional processing time. In contrast, endometrial aspiration cytology (EAC) can provide a cellular sample quickly, with relatively little discomfort and without the need for extensive instrumentation. Previous studies have described useful diagnostic concordance between EAC and histopathology, particularly for hyperplastic and malignant lesions [2-5].

Direct sampling of the endometrial cavity also offers a theoretical advantage over exfoliative cervicovaginal cytology because the material is obtained from the site of interest, usually yielding better-preserved and more abundant endometrial cells. The cytological appearance of proliferative and secretory endometrium, hyperplasia, atypia, and carcinoma can often be recognized when cellularity and preservation are adequate [4,6,7]. Nevertheless, the inability of cytology to assess gland-to-stroma relationships and invasion means that it cannot fully replace histology.

The present study was undertaken to evaluate the diagnostic accuracy of EAC compared with histopathology in women presenting with AUB at a tertiary care hospital. In addition to the primary diagnostic performance measures, the study examined the relationship of age, clinical bleeding pattern, ultrasonographic endometrial thickness, and cytological category with the final histopathological diagnosis.

## Materials and methods

This prospective cross-sectional study was conducted from January 2024 to December 2025 in the Department of Pathology, N.K.P. Salve Institute of Medical Sciences & Research Centre and Lata Mangeshkar Hospital, Nagpur, India. Data collection was performed from May 2024 to August 2025. Consecutive participants meeting the stated inclusion and exclusion criteria were enrolled by convenience sampling. Institutional Ethics Committee approval was obtained before commencement of the study, and written informed consent was obtained from all participants.

The minimum sample size was estimated using the following formula:

$n = Z^2_{(1-\alpha/2)} \times P(100-P) / L^2$

With Z = 1.96 for a 95% confidence level, an anticipated proportion (P) of 96% derived from the reference study by Vennila and Thangalakshmi [8], and an absolute precision (L) of 5%, the calculated sample size was 59.0 and was rounded up to 60. During the study period, 100 eligible participants were enrolled and analyzed.

Study population

Women presenting with symptomatic, clinically diagnosed AUB were eligible for inclusion. Pregnant women and women with a known structural genital cause of AUB identified before enrollment, including leiomyoma, endometrial polyp, or adenomyosis, were excluded. Clinical information included age and the presenting AUB pattern. Menorrhagia (heavy menstrual bleeding) was defined as bleeding at regular menstrual intervals lasting more than seven days or exceeding 80 mL per cycle; metrorrhagia as bleeding at irregular intervals between otherwise normal menstrual periods; polymenorrhea as a menstrual cycle interval shorter than 21 days; menometrorrhagia as excessive, prolonged, and irregular uterine bleeding; oligomenorrhea as menstrual intervals longer than 35 days but shorter than six months; and postmenopausal bleeding as genital tract bleeding after 12 consecutive months of amenorrhea following natural menopause.

Cytological sampling and evaluation

Endometrial aspirate obtained during the clinical sampling episode was spread onto glass slides and immediately fixed in ethyl alcohol. Smears were stained with Papanicolaou (Pap) and hematoxylin and eosin (H&E) stains and evaluated by light microscopy (up to 400× magnification). Assessment included cellularity, arrangement of glandular cells, nuclear size and chromatin, cytoplasmic characteristics, stromal fragments, background material, atypia, and features suspicious for malignancy. Endocervical cells, when present, were documented. Proliferative-phase cytology was identified by crowded/tubular glandular sheets with nuclear stratification; secretory-phase cytology by relatively uniform glandular cells in loose honeycomb sheets with cytoplasmic vacuolation; atypical hyperplasia by glandular crowding/overlap with cytological atypia; suspicious/malignant cytology by hypercellularity with enlarged hyperchromatic pleomorphic nuclei and scant cytoplasm; and granulomatous inflammation by epithelioid cell clusters in an inflammatory/stromal background.

Cytological diagnoses were grouped as proliferative endometrium, secretory endometrium, simple glandular hyperplasia without atypia, simple glandular hyperplasia with atypia, suspicious/malignant, or granulomatous. The corresponding histopathological specimen was processed routinely and served as the reference standard. Histopathological diagnoses were categorized as proliferative endometrium, secretory endometrium, simple glandular hyperplasia without atypia, simple glandular hyperplasia with atypia/endometrioid intraepithelial neoplasia, endometrial carcinoma, or granulomatous pathology. Atypical hyperplasia/endometrioid intraepithelial neoplasia was defined by glandular crowding with reduced intervening stroma and cytological atypia, while endometrial carcinoma required malignant glandular proliferation with architectural complexity and cytological atypia on histopathology.

Statistical analysis

Data were entered and analyzed using Epi Info version 7 (Centers for Disease Control and Prevention, Atlanta, Georgia). Categorical variables were summarized as n (%). Associations of age group, clinical presentation, endometrial thickness, and cytological category with the three-level histopathological classification (benign, premalignant, and malignant) were assessed using Pearson's chi-square tests, and the complete observed contingency counts were reported. A two-sided p-value below 0.05 was considered statistically significant. Diagnostic performance was recalculated directly from the 2×2 EAC-versus-histopathology table recorded in the source dataset (true positive = 45, false positive = 6, false negative = 5, true negative = 44) and expressed as sensitivity, specificity, positive predictive value (PPV), negative predictive value (NPV), and overall accuracy. Ninety-five percent confidence intervals for these proportions were calculated using the Wilson method.

## Results

A total of 100 women were evaluated. The study population was concentrated in the middle and later reproductive years: 38 (38%) were aged 41-50 years and 32 (32%) were aged 31-40 years. Menorrhagia was the most common presenting pattern found in 36 (36%), followed by metrorrhagia in 20 (20%) participants. On ultrasonography, 46 (46%) had an endometrial thickness of 5-10 mm, while 38 (38%) had a thickness greater than 10 mm (Table 1).

**Table 1:** Clinical and ultrasonographic profile of the study participants (n = 100).

Characteristic	Category	n (%)
Age group (years)	21-30	18 (18%)
	31-40	32 (32%)
	41-50	38 (38%)
	51-60	9 (9%)
	>60	3 (3%)
Clinical presentation	Menorrhagia	36 (36%)
	Metrorrhagia	20 (20%)
	Polymenorrhea	14 (14%)
	Postmenopausal bleeding	12 (12%)
	Menometrorrhagia	10 (10%)
	Oligomenorrhea	8 (8%)
Endometrial thickness	<5 mm	16 (16%)
	5-10 mm	46 (46%)
	>10 mm	38 (38%)

Normal cyclical endometrium accounted for most cytological diagnoses. Secretory endometrium was identified in 38 (38%) cases and proliferative endometrium in 35 (35%). Hyperplasia with atypia was reported cytologically in 11 (11%), hyperplasia without atypia in eight (8%), suspicious/malignant cytology in seven (7%), and granulomatous changes in one (1%). Histopathology showed a closely comparable distribution, with secretory endometrium in 37 (37%) and proliferative endometrium in 34 (34%). Histopathology identified simple glandular hyperplasia without atypia in 10 (10%), hyperplasia with atypia in nine (9%), endometrial carcinoma in nine (9%), and granulomatous pathology in one (1%) (Table 2). Representative proliferative- and secretory-phase cytomorphology images are shown in Figure 1 and Figure 2, respectively. Cytological features of glandular hyperplasia with atypia and granulomatous inflammation are illustrated in Figure 3 and Figure 4, respectively. A representative malignant case with paired cytology and histopathology is shown in Figure 5 and Figure 6, respectively.

**Table 2:** Distribution of endometrial aspiration cytology (EAC) and histopathological diagnoses.

Diagnosis	EAC, n (%)	Histopathology, n (%)
Proliferative endometrium	35 (35%)	34 (34%)
Secretory endometrium	38 (38%)	37 (37%)
Simple glandular hyperplasia without atypia	8 (8%)	10 (10%)
Simple glandular hyperplasia with atypia	11 (11%)	9 (9%)
Suspicious/malignant cytology/endometrial carcinoma	7 (7%)	9 (9%)
Granulomatous	1 (1%)	1 (1%)

**Figure 1:**
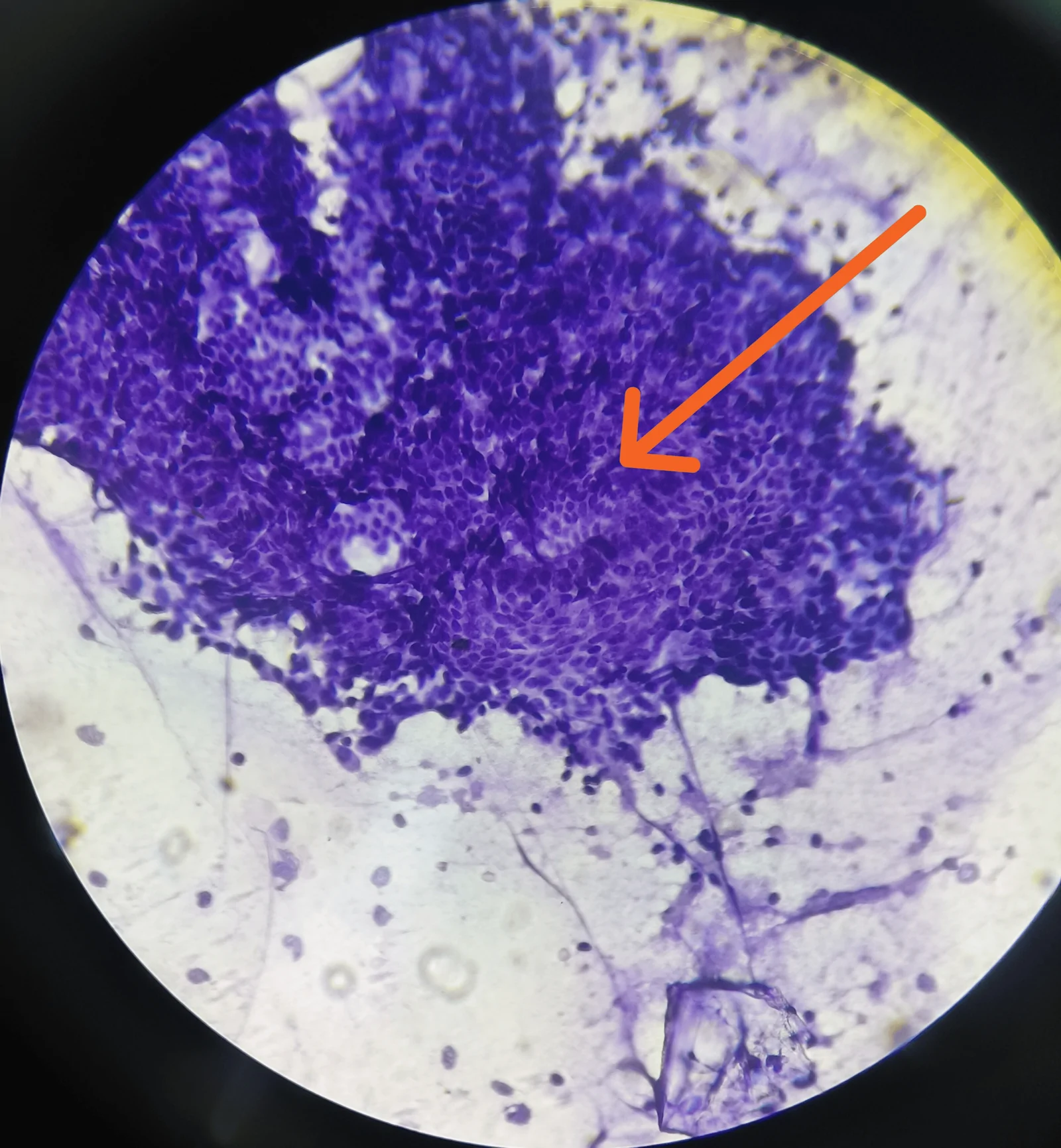
Proliferative-phase endometrial cytology. The smear shows crowded sheets and clusters of glandular cells with nuclear stratification and mild nuclear enlargement; stromal fragments are present in a relatively clean background. The arrow indicates a representative crowded glandular cell sheet showing nuclear stratification.

**Figure 2:**
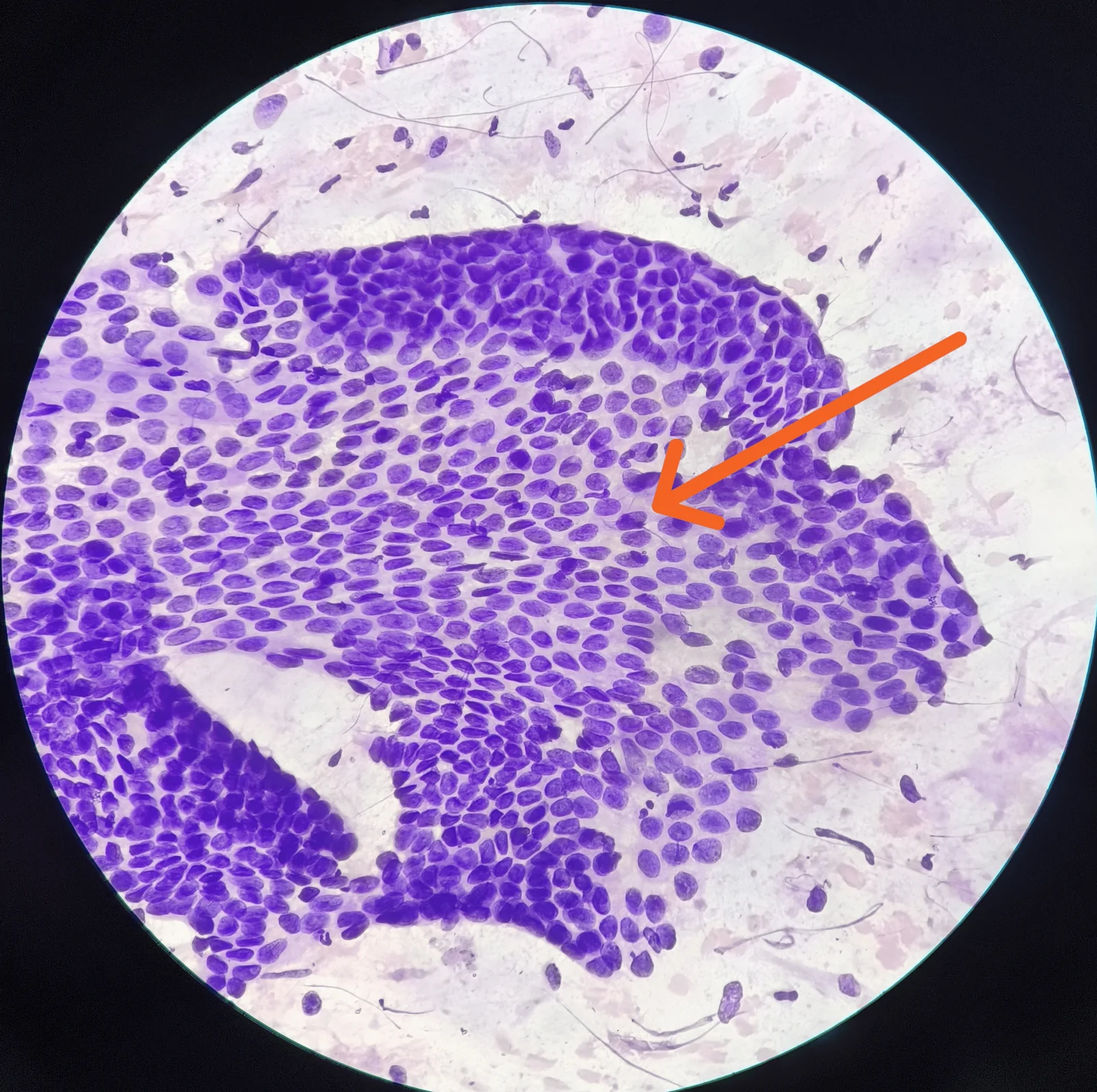
Secretory-phase endometrial cytology. Glandular cells are arranged in a loose honeycomb pattern with relatively uniform nuclei and cytoplasmic vacuolation. The arrow indicates a representative glandular sheet showing the honeycomb arrangement and cytoplasmic vacuolation.

**Figure 3:**
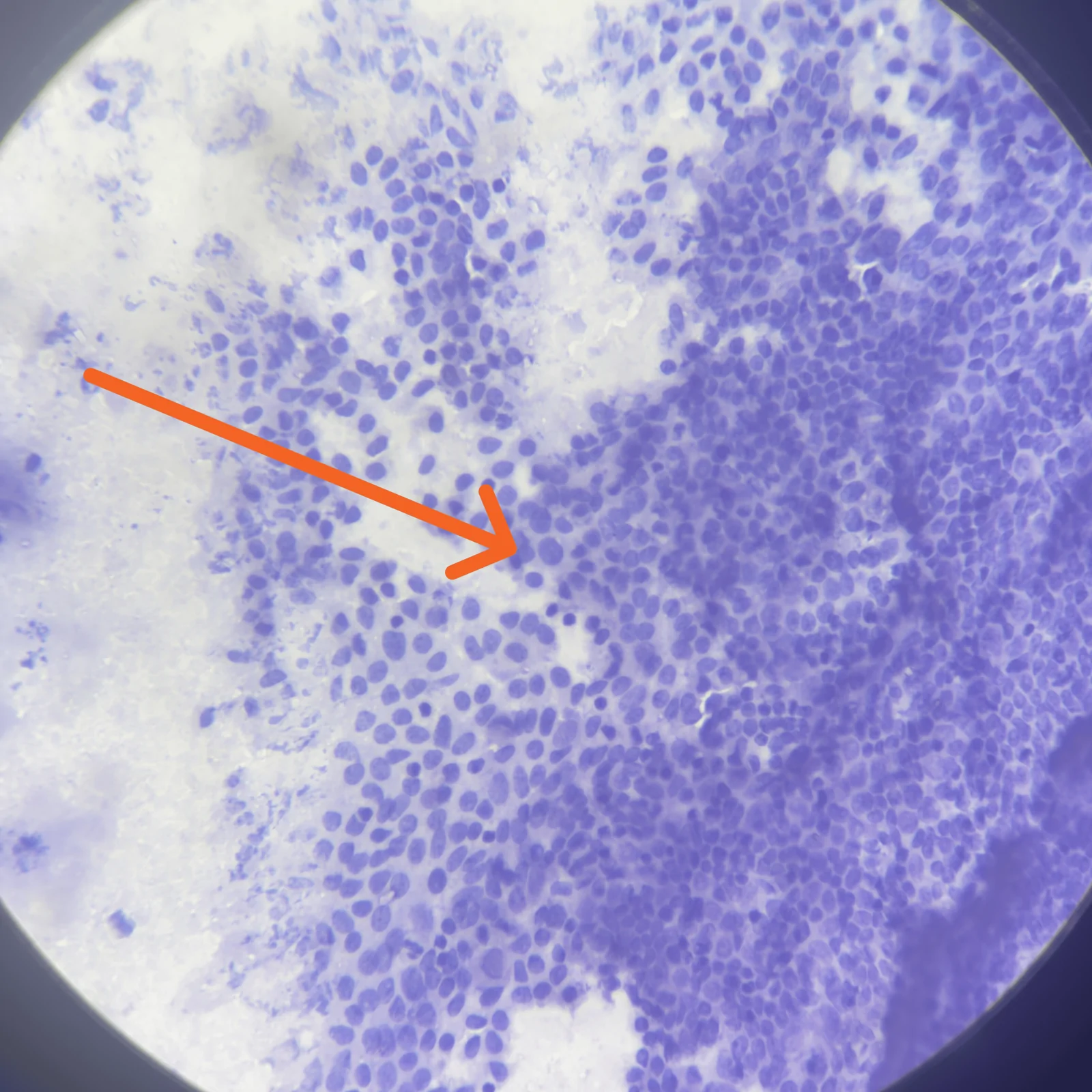
Endometrial glandular hyperplasia with atypia on cytology. Crowded and overlapping glandular cells show enlarged, mildly atypical nuclei with moderate cytoplasm. The arrow indicates a representative crowded, overlapping atypical glandular cell cluster.

**Figure 4:**
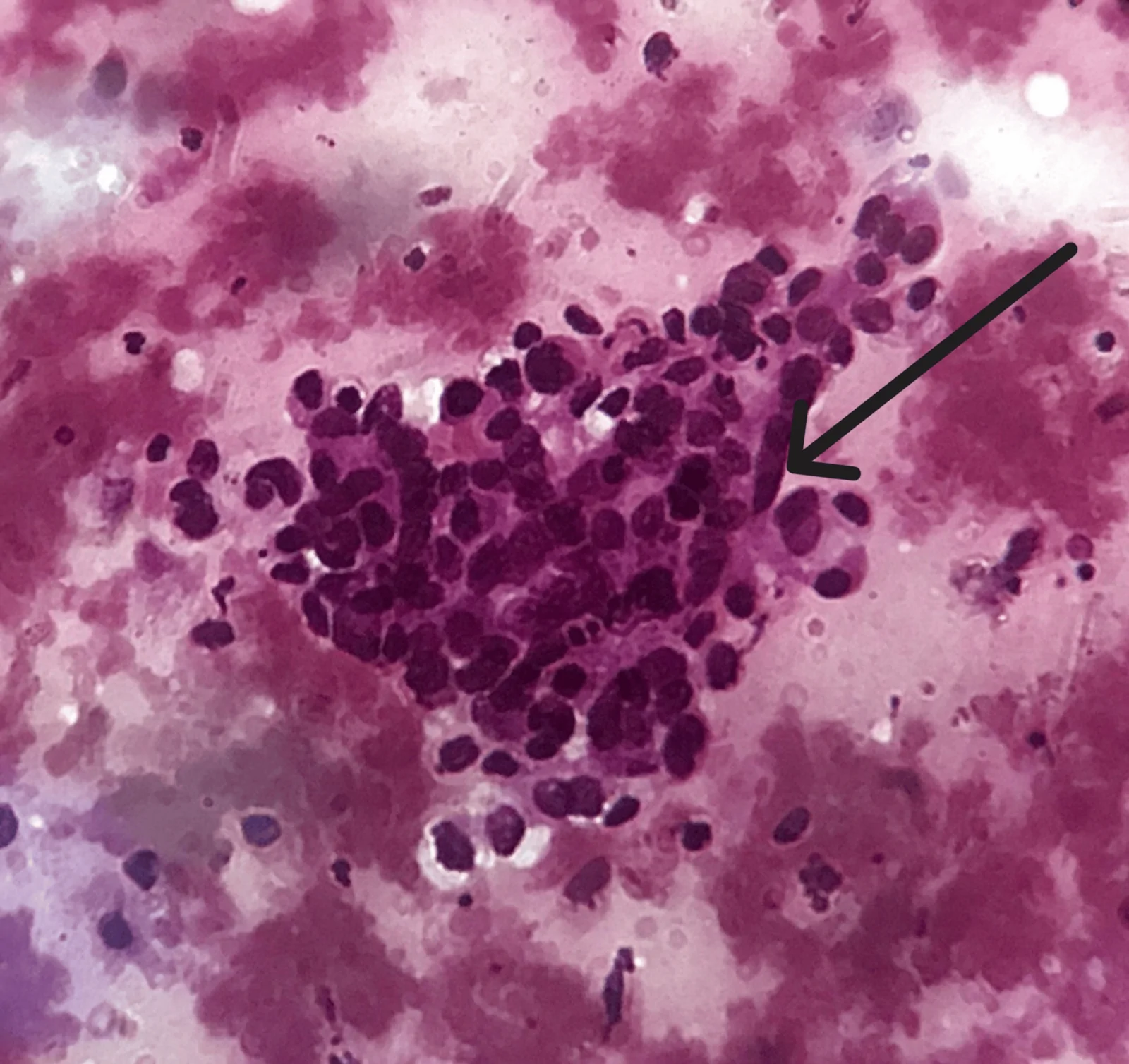
Granulomatous cytology. The background contains small stromal fragments with occasional clusters of epithelioid cells, in keeping with granulomatous inflammation. The arrow indicates a representative cluster of epithelioid cells.

**Figure 5:**
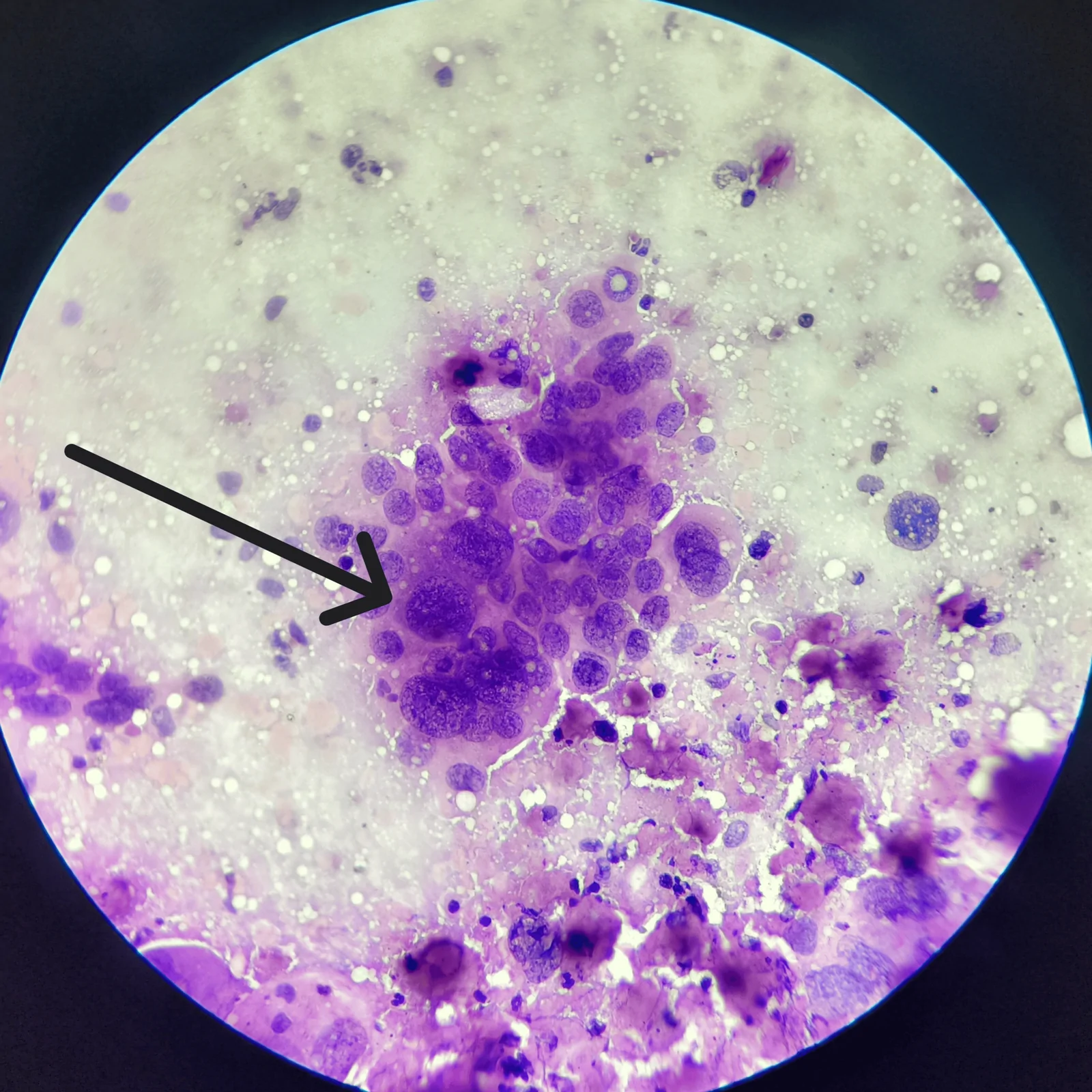
Endometrial malignancy. Hypercellular cytology smear with sheets and clusters of glandular cells showing enlarged, hyperchromatic nuclei and scant cytoplasm. The arrow highlights a representative malignant glandular cell cluster.

**Figure 6:**
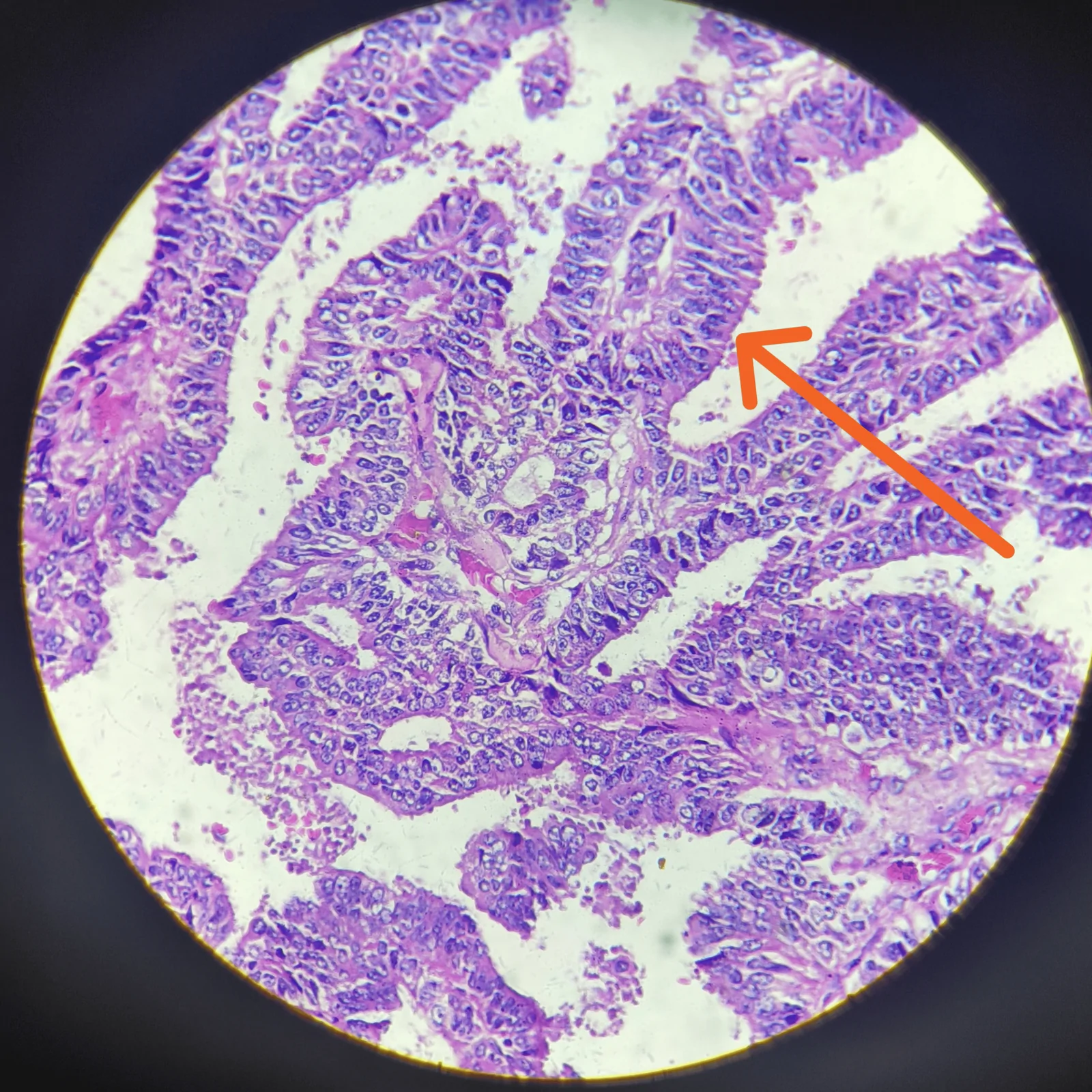
Endometrial malignancy. Histopathology demonstrates architecturally complex endometrial glands with anisonucleosis and pleomorphism. The arrow indicates a representative architecturally complex malignant gland.

The relationship between the clinicoradiological variables and histopathological category was statistically significant. Premalignant and malignant lesions increased with advancing age (Pearson chi-square = 33.165, df = 8, p < 0.001); among women aged 41-50 years, 10/38 (26.3%) had premalignant and 4/38 (10.5%) had malignant pathology. Postmenopausal bleeding showed the strongest malignant association, with 6/12 (50.0%) women having malignant histopathology (Pearson chi-square = 32.987, df = 10, p < 0.001). Endometrial thickness was also associated with final diagnosis: among 38 women with an endometrium thicker than 10 mm, 12/38 (31.6%) had premalignant and 7/38 (18.4%) had malignant lesions (Pearson chi-square = 16.011, df = 4, p = 0.003). Cytological categorization correlated strongly with histopathology (Pearson chi-square = 132.237, df = 4, p < 0.001) (Table 3).

**Table 3:** Association of clinicoradiological and cytological variables with histopathological diagnosis. Values are numbers (% within rows). The observed counts shown were used in the Pearson chi-square calculations.

Variable	Category	Benign, n (%)	Premalignant, n (%)	Malignant, n (%)	Pearson χ² (df); p
Age group (years)	21-30	17 (94.4%)	1 (5.6%)	0 (0.0%)	33.165 (df=8); p<0.001
	31-40	28 (87.5%)	4 (12.5%)	0 (0.0%)	
	41-50	24 (63.2%)	10 (26.3%)	4 (10.5%)	
	51-60	3 (33.3%)	3 (33.3%)	3 (33.3%)	
	>60	0 (0.0%)	1 (33.3%)	2 (66.7%)	
Clinical presentation	Menorrhagia	30 (83.3%)	5 (13.9%)	1 (2.8%)	32.987 (df=10); p<0.001
	Metrorrhagia	12 (60.0%)	6 (30.0%)	2 (10.0%)	
	Polymenorrhea	12 (85.7%)	2 (14.3%)	0 (0.0%)	
	Postmenopausal bleeding	4 (33.3%)	2 (16.7%)	6 (50.0%)	
	Menometrorrhagia	8 (80.0%)	2 (20.0%)	0 (0.0%)	
	Oligomenorrhea	6 (75.0%)	2 (25.0%)	0 (0.0%)	
Endometrial thickness	<5 mm	15 (93.8%)	1 (6.2%)	0 (0.0%)	16.011 (df=4); p=0.003
	5-10 mm	38 (82.6%)	6 (13.0%)	2 (4.3%)	
	>10 mm	19 (50.0%)	12 (31.6%)	7 (18.4%)	
Cytological category	Benign	66 (94.3%)	4 (5.7%)	0 (0.0%)	132.237 (df=4); p<0.001
	Hyperplasia	6 (30.0%)	14 (70.0%)	0 (0.0%)	
	Malignant/suspicious	0 (0.0%)	1 (10.0%)	9 (90.0%)	

The 2×2 diagnostic table contained 45 true-positive, six false-positive, five false-negative, and 44 true-negative results. Accordingly, EAC achieved a sensitivity of 45/50 (90.0%), specificity of 44/50 (88.0%), PPV of 45/51 (88.2%), NPV of 44/49 (89.8%), and overall diagnostic accuracy of 89/100 (89.0%). The corresponding Wilson 95% confidence intervals are shown in Table 4.

**Table 4:** Diagnostic performance of endometrial aspiration cytology compared with histopathology.

Parameter	n/N (%)	95% confidence interval
Sensitivity	45/50 (90.0%)	78.6-95.7
Specificity	44/50 (88.0%)	76.2-94.4
Positive predictive value	45/51 (88.2%)	76.6-94.5
Negative predictive value	44/49 (89.8%)	78.2-95.6
Diagnostic accuracy	89/100 (89.0%)	81.4-93.7

## Discussion

The present study examined EAC as a practical diagnostic test in 100 women presenting with AUB and found a close overall relationship between the cytological impression and histopathological diagnosis. The diagnostic indices were clinically useful, with sensitivity of 45/50 (90.0%), specificity of 44/50 (88.0%), and overall accuracy of 89/100 (89.0%). These findings support the role of EAC as an early diagnostic step rather than as a replacement for histopathology.

The age distribution was characteristic of a tertiary-care AUB population. Seventy of 100 participants (70.0%) were between 31 and 50 years of age, and the single largest group was 41-50 years with 38/100 (38.0%). This pattern is biologically plausible: the perimenopausal transition is often accompanied by anovulatory cycles and variable estrogen-progesterone exposure, which can produce irregular shedding and sustained endometrial proliferation. In the present series, advancing age was also associated with a higher proportion of premalignant and malignant pathology (p < 0.001), reinforcing the need for a lower threshold for endometrial assessment in older women with new or persistent bleeding [9,10].

Menorrhagia was the most frequent symptom, affecting 36/100 (36.0%) participants. Most women with menorrhagia had benign histology, whereas postmenopausal bleeding showed a very different pattern: 6/12 (50.0%) women with postmenopausal bleeding had malignant histopathology. The association between bleeding pattern and histopathology was significant (p < 0.001). Although the absolute numbers were small, this is an important clinical signal and is consistent with the established principle that postmenopausal bleeding warrants prompt exclusion of endometrial malignancy [9-11].

Ultrasonographic endometrial thickness also helped separate lower- and higher-risk groups. Fifteen of 16 women with an endometrium thinner than 5 mm had benign histology (15/16, 93.8%), while premalignant and malignant lesions were concentrated among women with a thickness greater than 10 mm (12/38 (31.6%) and 7/38 (18.4%), respectively). The association was significant (p = 0.003). Imaging therefore appears most useful when interpreted together with the clinical presentation and cytological findings, rather than as an isolated test.

The morphological distribution on EAC closely mirrored histopathology. Secretory endometrium was the most common pattern on both cytology and HPE, followed by proliferative endometrium. The small differences in counts between the two methods are expected because cytology evaluates dispersed cells and tissue fragments, whereas histology preserves architecture. This distinction becomes especially important in hyperplasia, where glandular crowding, gland-to-stroma ratio, and complex architecture can materially affect classification.

Previous investigators have reported similarly useful performance for EAC. Kaur et al. found high sensitivity for benign lesions, hyperplasia, and malignancy, with specificity generally ranging from 90% to 100% [2]. Handa et al. also reported good cytohistological agreement and noted that malignant lesions were often recognized more reliably than subtle cyclical abnormalities [4]. In the study by Dongre and Maimoon, EAC achieved 90.66% sampling adequacy, with a sensitivity of 88.7% for benign conditions and 100% for malignant conditions [5]. The present study's sensitivity of 45/50 (90.0%) and overall accuracy of 89/100 (89.0%) therefore fall within the clinically useful range reported in the literature.

The value of EAC is not limited to a single accuracy statistic. The procedure is rapid, relatively inexpensive, and suitable for outpatient practice. A well-prepared smear can provide an immediate sense of whether the endometrium is cyclical, hyperplastic, atypical, inflammatory, or frankly malignant. This can help prioritize further investigation and management, particularly in high-volume or resource-constrained settings. Direct endometrial sampling also yields better-preserved material than relying on shed endometrial cells in routine cervicovaginal preparations [5,12].

At the same time, the limitations of cytology are intrinsic to the technique. Scant cellularity and sampling error may cause false-negative results, while overlapping cytomorphological features can lead to overinterpretation. More importantly, EAC cannot reliably assess tissue architecture, depth of invasion, or focal lesions such as polyps. Histopathology therefore remains necessary whenever cytology is atypical or suspicious, when symptoms persist despite a benign smear, or when imaging and clinical findings raise concern. Studies comparing aspiration with curettage and investigations of liquid-based endometrial cytology similarly emphasize that cytology is best viewed as part of a diagnostic pathway rather than a stand-alone definitive test [13-15].

Limitations

This was a single-center study with a sample of 100 participants, and the findings may not be directly generalizable to other populations. The study used convenience sampling, which can introduce selection bias. Cytological interpretation is operator-dependent, and the source thesis did not provide a separate analysis of sampling adequacy or interobserver variability. Several cells in the clinicopathological contingency tables had small expected frequencies; therefore, the Pearson chi-square association results should be interpreted cautiously. In addition, the source dataset contained a separate binary positive/negative classification for the diagnostic 2×2 analysis, but the exact threshold used to define binary positivity was not explicitly documented in the thesis. Future studies should define that threshold prospectively. Finally, cytology cannot substitute for architectural assessment when hyperplasia, atypia, or malignancy is suspected.

## Conclusions

EAC demonstrated good agreement with histopathology and useful diagnostic performance in women presenting with AUB. The test was particularly helpful for identifying hyperplastic and suspicious/malignant patterns, while age, postmenopausal bleeding, and increased endometrial thickness provided additional clinical context for risk stratification. As EAC is rapid, minimally invasive, and inexpensive, it can serve as a practical screening and preliminary diagnostic tool in both routine and resource-limited settings. Histopathological examination should remain the definitive method for confirmation and classification of premalignant and malignant endometrial disease.

## References

[1] Munro MG, Critchley HO, Fraser IS (2018). The two FIGO systems for normal and abnormal uterine bleeding symptoms and classification of causes of abnormal uterine bleeding in the reproductive years: 2018 revisions. Int J Gynaecol Obstet.

[2] Kaur N, Chahal JS, Bandlish U, Kaul R, Mardi K, Kaur H (2014). Correlation between cytological and histopathological examination of the endometrium in abnormal uterine bleeding. J Cytol.

[3] Singh P (2018). Abnormal uterine bleeding- evaluation by endometrial aspiration. J Midlife Health.

[4] Handa U, Bansal C, Aggarwal P, Huria A, Mohan H (2018). Diagnostic utility of endometrial aspiration cytology in women with abnormal uterine bleeding. J Midlife Health.

[5] Dongre TA, Maimoon SA (2023). Endometrial aspiration cytology: a cross-sectional comparative study of its efficacy and sensitivity in diagnosing gynecological disorders. Cureus.

[6] Fujiwara H, Takahashi Y, Takano M (2015). Evaluation of endometrial cytology: cytohistological correlations in 1,441 cancer patients. Oncology.

[7] Baxi SN, Panchal NS (2015). Histopathology-like categories based on endometrial imprint cytology in dysfunctional uterine bleeding. J Cytol.

[8] Vennila M, Thangalakshmi Thangalakshmi (2021). Value of endometrial aspiration cytology in assessing the endometrial status in symptomatic peri and postmenopausal women and its histological correlation with fractional curettage. Int J Reprod Contracept Obstet Gynecol.

[9] Marnach ML, Laughlin-Tommaso SK (2019). Evaluation and management of abnormal uterine bleeding. Mayo Clin Proc.

[10] Jain V, Munro MG, Critchley HO (2023). Contemporary evaluation of women and girls with abnormal uterine bleeding: FIGO Systems 1 and 2. Int J Gynaecol Obstet.

[11] O'Flynn H, Ryan NA, Narine N, Shelton D, Rana D, Crosbie EJ (2021). Diagnostic accuracy of cytology for the detection of endometrial cancer in urine and vaginal samples. Nat Commun.

[12] Jadhav MV, Phatke AS, Kadgi NV, Rane SR, Kulkarni KK (2016). Endometrial aspiration cytology in gynecological disorders. J Cytol.

[13] Norimatsu Y, Yanoh K, Hirai Y, Kurokawa T, Kobayashi TK, Fulciniti F (2020). A diagnostic approach to endometrial cytology by means of liquid-based preparations. Acta Cytol.

[14] Wang Q, Wang Q, Zhao L (2019). Endometrial cytology as a method to improve the accuracy of diagnosis of endometrial cancer: case report and meta-analysis. Front Oncol.

[15] Hwang WY, Suh DH, Kim K, No JH, Kim YB (2021). Aspiration biopsy versus dilatation and curettage for endometrial hyperplasia prior to hysterectomy. Diagn Pathol.

